# Infusion of autologous bone marrow mononuclear cells leads to transient reduction in proteinuria in treatment refractory patients with Idiopathic membranous nephropathy

**DOI:** 10.1186/1471-2369-14-262

**Published:** 2013-12-01

**Authors:** Upal Sengupta, Vinod Kumar, Ashok K Yadav, Neelam Marwaha, Harbir S Kohli, Vinay Sakhuja, Vivekanand Jha

**Affiliations:** 1 Department of Nephrology, Postgraduate Institute of Medical Education and Research, Chandigarh, India; 2 Department of Tranfusion Medicine, Postgraduate Institute of Medical Education and Research, Chandigarh, India; 3 George Institute for Global Health, New Delhi, India

## Abstract

**Background:**

The current treatment options for idiopathic membranous nephropathy (IMN) carry significant toxicity. In this prospective, observational pilot study, we used single time infusion of bone marrow derived autologous mononuclear cells (MNCs) in adult patients with treatment refractory IMN.

**Methods:**

Twelve patients of biopsy proven IMN who had failed a cyclical 6-month regimen of steroid and cyclophosphamide were enrolled in the study. Bone-marrow was harvested from the iliac crest and underwent processing to isolate MNCs. Cells were counted and subjected to viability testing before being infused through a peripheral vein on the same day. After the infusion, subjects were followed up monthly for the next six months. Supportive treatment including angiotensin antagonists and statins was continued throughout the study period.

**Result:**

The proteinuria, serum albumin and creatinine values at entry were 2.97 ± 0.6 gm/1.73 m^2^/d, 2.27 ± 1.1 gm/l and 0.9 ± 0.8 mg/dl respectively. There was a reduction in proteinuria (p < 0.0001), and increase in serum albumin (p = 0.001) at 1 month, with 64% of the subjects showing >50% reduction in proteinuria. However, the response was ill sustained. At 6 months, only 2 patients had >50% reduction. Serum creatinine remained stable throughout the study period. No infusion related side effects were noted.

**Conclusion:**

Autologous mononuclear cell infusion leads to transitory reduction in proteinuria and improvement in serum albumin in treatment refractory IMN. This effect, however, is transient. Whether this can be overcome by repeated infusion of cultured mesenchymal cells needs to be investigated.

## Background

Idiopathic membranous nephropathy (IMN), the major cause of nephrotic syndrome in adults, is an autoimmune disorder. The recent discovery of phospholipase A2 receptor (PLA2R) as the major target antigen and the association of anti-PLA2R with disease activity has made the role of specific dysregulation in the immune system clearer [1]. IMN Patients typically present with nephrotic syndrome; about a third remit spontaneously but of the remainder, about 50% progress to ESRD by 10 years without treatment. Treatment involves the use of immunosuppressive drugs such as high dose-steroids and alkylating agents or calcineurin inhibitors [2-4]. More recently, the monoclonal anti-CD20 antibody rituximab has shown promise [5]. Treatment is not successful in all cases, associated with significant short and long-term side effects and in the case of the last agent, expensive. Further, identification of high-risk patients who would benefit from such a therapy is at best empirical and approximate. Availability of relatively nontoxic therapy that could be applied to most patients without fear of major adverse effects holds a lot of attraction.

In recent years, the immunomodulatory property of stem cells has received attention. The first evidence of such an effect became evident from the therapeutic benefit in refractory graft versus host disease following hematopoietic stem cell transplantation [6]. The proposed underlying mechanism is by elaboration of soluble anti-inflammatory mediators as well as direct suppression of T-cells and dendritic cells [7]. There is encouraging progress in the use of these cells in animal models of SLE, multiple sclerosis and Crohn’s disease [8]. Their use in human subjects, however, is limited. This pilot study was conducted as a proof-of-concept study as well as to establish the safety profile of one-time infusion of autologous mononuclear cells in adults with treatment refractory IMN.

## Methods

Twelve patients were included in this pilot prospective observational study on the basis of following inclusion criteria: age ≥ 16 years, biopsy proven IMN, already received and failed a 6 months regimen of cyclical monthly steroid and cyclophosphamide and/or CNI, off all immunosuppression for at least six months, nephrotic syndrome (defined as proteinuria ≥3.5 g/1.73 m^2^/d or ≥2 g/d along with serum albumin <2.5 g/dl, edema, and hyperlipidemia) and on optimal therapy with angiotensin converting enzyme inhibitors and/or angiotensin receptor blockers.

We excluded patients with systemic illness, malignancy, diabetes, hepatitis B surface antigen positivity, or renal vein thrombosis, pregnant patients, patient who failed to give consent, cases with secondary membranous nephropathy, those with nephrotic syndrome < 1 year, and patients with eGFR <30 ml/min

Clearance was obtained from the Institute Ethics Committee and the Institute Committee on Stem Cell Research and Therapy (Approval No IC-SCRT-18/2010/3576). All subjects were explained about the exact nature of the interventions, its potential benefits and harms and provided written consent.

Mononuclear cells (MNCs) were processed from harvested bone marrow of individual patients. Bone marrow aspirations were performed under sterile conditions under local anesthesia from right posterior superior iliac spine. Collected bone marrow was processed according to recommended level of sterility and biosafety precautions. After processing, obtained cell counts as well as viability were checked. About fifty μl of the final product was kept for flowcytometric estimation of CD45, CD34 and CD105 positivity and rest of the product was diluted to a final volume of 10 ml. Cells were administered intravenously over 30 minutes. Patients were kept overnight in the hospital and monitored for any delayed adverse reactions.

All subjects were followed up once a month for a total period of six months. Supportive therapy including ACEI ± ARBs, statins, antihypertensive and diuretics were continued. The primary endpoint was change in proteinuria. 

Statistical analysis was performed using IBM SPSS v 20.0 and GraphPad Prism v 4.0. Proteinuria, serum albumin and serum creatinine in the six consecutive monthly visits were compared using non-parametric Friedman test with Dunn’s post hoc analysis to identify the visits between which there was a statistically significant difference. The proteinuria and serum albumin at the end of first month were correlated with the infusate parameters as well as other baseline values using Spearman bivariate correlation test. A p value of less that 0.05 was considered statistically significant

## Results

A total of 12 subjects were recruited. One patient was lost to follow up after the first visit following the procedure, and 11 were available for analysis. Table 1 shows the baseline characteristics of the subjects. Out of the 11 patients, there were six males, and the age range was 18–45 years. All the patients were on ACE inhibitor or ACE inhibitor with combination with ARBs. All the subjects had already received and failed a six-month course of cyclical monthly steroid and cyclophosphamide therapy. Four patients had also received tacrolimus for 3–4 months. All subjects had edema and were receiving diuretics at the time of inclusion. Nine (75%) patients were receiving HMG-CoA inhibitor therapy. Two had past episodes of venous thrombosis, with one having had two episodes. Of all the patients recruited, one did not satisfy the proteinuria/serum albumin criteria but had experienced two episodes of venous thrombosis and was symptomatic and hence was considered to be a candidate for treatment.

**Table 1:** Patient characteristics

**Parameter**	**Value**
Number of cases	11
Age (years)	29.36 ± 3.31
Gender ratio (M:F)	6:5
Duration of disease (months)	23 ± 4
Edema	11 (100)
Hypertension	10 (91)
Proteinuria (gm/day)	2.28 ± 0.4
Proteinuria (gm/1.73m^2^/day)	2.97 ± 0.6
Serum albumin (g/dl)	2.27 ± 1.1
Serum creatinine (mg/dl)	0.9 ± 0.8
Hypoalbuminemia	11 (100)
Hyperlipidemia	11 (100)
Previous treatment	
Cyclophosphamide/steroids	11 (100)
Tacrolimus	4 (36)
Concomitant therapy	
ACEI only	4 (36)
ACEI + ARB	7 (64)
Diuretics	11 (100)
Statins	9 (82)

The volume of the bone marrow aspirate was 162.1 ± 25.1 ml. The number of the injected mononuclear cells ranged between 8.51 × 10^7^ and 2.32 × 10^8^. The cells exhibited high (96.27 ± 1.8%) viability. CD34 positivity was seen in 0.38 ± 0.08% cells.

**Figure 1:**
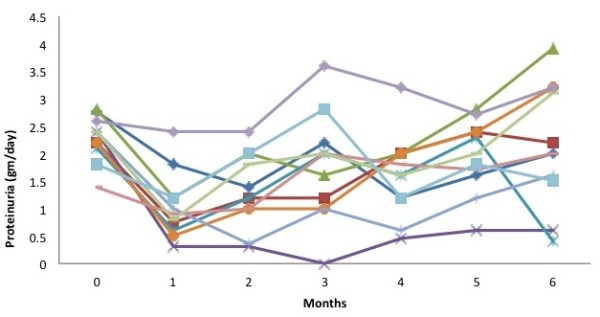
Baseline and follow-up values of proteinuria.

**Figure 2:**
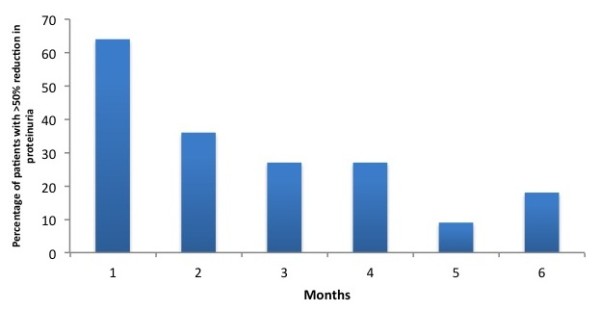
Proportion of subjects with greater than 50% decrease in proteinuria.

**Figure 3:**
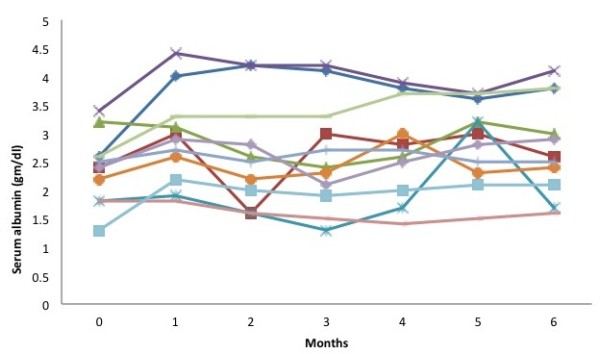
Baseline and follow-up values of serum albumin.

**Figure 4:**
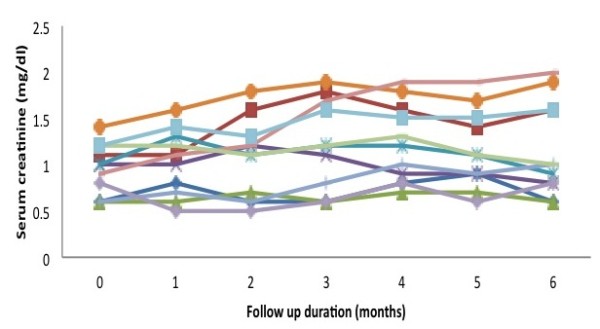
Baseline and follow-up values of serum creatinine.

All subjects showed a reduction in proteinuria ranging from 8-85% (Figure 1). The value decreased to 1.0 ± 0.61 g/day (p < 0.0001) at one month and 1.33 ± 0.7 g/day (p = 0.001) at 2 months. None of the subjects underwent complete remission. The proteinuria came back to 1.8 ± 1 g/day At 3 months and 2.15 ± 1.1 g/day at 6 months (p > 0.05). About 64% of the subjects showed more than 50% decrease in proteinuria in the first month, which decreased to 9% in the fifth month of follow-up (Figure 2). This was associated with improvement in serum albumin to the tune of 6-70% at 1 month (2.27 ± 0.57 d/dl vs 2.9 ± 0.8 g/dl, p = 0.001, Figure 3). However, the response was ill sustained, the proteinuria again increased and the serum albumin declined in majority of the cases. There was no effect of this therapy on serum creatinine (Figure 4).

The 24-hour urinary protein excretion at the first month was not significantly correlated with any of the infusate parameters (i.e. aspirate volume, number of cells, percentage of MNCs or the viability of the cells), baseline proteinuria or serum albumin values.

None of the patients experienced any infusion related immediate adverse effects. Over six months of follow-up, there was no report of any adverse events.

## Discussion and conclusions

Cell-based therapies are being tried for several refractory diseases, including immunologically mediated diseases such as graft-versus-host disease, systemic lupus erythematosus, rheumatoid arthritis and inflammatory bowel disease, and for tolerance induction following kidney transplantation [9-11]. Systemic administration of BM-MNCs has been shown to be beneficial in coronary artery disease, cardiomyopathy, diabetes mellitus and peripheral vascular disease [12-14]. How exactly these cells exert their favorable influence in these conditions is not known, but postulated mechanisms include paracrine and/or immunomodulatory properties of progenitor cells. Several cell types present in peripheral blood have immunomodulatory properties, including the hematopoietic and mesenchymal stem cells (MSC), dendritic cells and various T-cell subsets.

IMN has all the attributes of the prototype autoimmune disease, with the uniform presence of immune deposits in the biopsy specimens, recent discovery of circulating autoantibodies (anti-PLA2R) and response to immunosuppressive therapies. Although the discovery of anti-PLA2R antibody has been an interesting landmark in understanding the disease pathogenesis, there has been no major progress in the treatment of this entity [1]. Even in the present time, treatment with steroid and alkylating agents or calcineurin inhibitors remains the gold standard. About 70% - 80% respond to such treatment, but both regimes carry significant toxicities [15]. As only a subset of patients with IMN show progressive renal dysfunction, ideally only these patients should receive therapy to alter disease course. Currently, however, there are no reliable markers to identify such patients at presentation. The current strategy is to wait and reserve immunosuppressive treatment for patients with persistent heavy proteinuria and/or declining renal function. There is some evidence to suggest that earlier treatment is associated with better response rates. In an RCT where patients in control group were allowed to cross over after 2 years, the remission rate in this group was inferior compared to those in the intervention group [3].

This preliminary study shows that use of autologous bone marrow MNCs is safe and associated with transient improvement in proteinuria in patients with IMN refractory to conventional treatment. Although the proteinuria was relatively modest by western standards, all patients had clinical manifestations of nephrotic state, most had severe persistent hypoalbuminemia, edema, hyperlipidemia and/or complications related to nephrotic syndrome. We believe that the proteinuria might have come down secondary to severe hypoalbuminemia. This is a common observation in the vegetarian Indian subjects. The edema decreased and serum albumin improved after cell therapy, albeit transiently.

As most of the injected MNCs get trapped into pulmonary microcirculation upon systemic injection [16], it is unlikely that the administered cells would have actually reached the kidneys in significant numbers and repaired the damage by transdifferentiation. It is more likely that cytokines secreted by the cells after injection might be responsible for any benefit. This could also explain the transient treatment effect.

While the improvement in proteinuria as well as serum albumin one month post-procedure in a significant proportion of patients was encouraging, the response was ill sustained. This raises the question whether administration of a larger dose or repeated injections could provide a more sustained benefit. Repeated bone marrow aspirations, however, are not practical. Use of autologus bone-marrow derived MSC, as is being currently tried for immunomodulation in several diseases including for inducing tolerance following kidney transplantation, might be a more suitable option for repeated administration, since these cells can be expanded and stored.

Our study provides the basis for further exploration of cell-based therapy approaches in this condition. Possible next steps could be use of cultured MSCs and/or cell free extracts from supernatants of MSC culture. Since levels of phospholipase A2 receptor reflect disease activity and have been shown to correlate with response, serial measurements of PLA2R levels would be useful in attributing causality to response.

In conclusion, we show in this pilot study that use of autologous bone marrow MNCs leads to transient decrease in proteinuria and improvement in hypoalbuminemia in IMN patients refractory to standard therapy. MNC infusion is safe in this condition. More studies are needed to explore the role of cell-based therapies in this condition.

## Competing interest

All the authors declare no conflict of interest.

The results presented in this paper have not been published previously in whole or part. This study was presented in the Annual Meeting of the American Society of Nephrology in 2012 at San Diego.

## Authors’ contributions

US: Selected and followed up patients, analyzed data and wrote the paper. VSK: Processed and characterized MNCs and helped in manuscript writing. AKY: Analyzed data and helped in manuscript writing. NM: Study design, quality control and cell viability studies. HSK: Patient selection follow up and data interpretation. VS: Study design, patient recruitment and follow up, study oversight. VJ: Conceptualized and designed the study protocol, oversaw the study conduct, reviewed and finalized the paper. All authors read and approved the final version of the manuscript.

## Pre-publication history

The pre-publication history for this paper can be accessed here:

http://www.biomedcentral.com/1471-2369/14/262/prepub
